# Reduced Sensorimotor, Working Memory, and Episodic Memory Abilities in Aging Female *FMR1* Premutation Carriers with and Without Fragile X-Associated Tremor/Ataxia Syndrome (FXTAS)

**DOI:** 10.3390/genes16111331

**Published:** 2025-11-04

**Authors:** Kristen McGatlin, Robin L. Shafer, Kathryn E. Unruh, Cassandra J. Stevens, Sophia G. Peterson, Richard M. Dubinsky, Andrea P. Lee, Flora Tassone, Randi J. Hagerman, Heather Bailey, Matthew W. Mosconi

**Affiliations:** 1 Department of Psychological Sciences, Kansas State University, Manhattan, KS 66506, USA; kmcgatlin@ksu.edu (K.M.); hbailey@ksu.edu (H.B.); 2 Kansas Center for Autism Research and Training, Life Span Institute, University of Kansas, Lawrence, KS 66045, USA; rshafer3@ku.edu (R.L.S.); katunruh@ku.edu (K.E.U.); cassandrastevens@ku.edu (C.J.S.); sgracepeterson@ku.edu (S.G.P.); 3 Department of Clinical Child Psychology, University of Kansas, Lawrence, KS 66045, USA; 4 Department of Neurology, University of Kansas Medical Center, Kansas City, KS 66160, USA; rdubinsk@kumc.edu (R.M.D.); alee19@kumc.edu (A.P.L.); 5 Medical Investigation of Neurodevelopmental Disorders (MIND) Institute, University of California Davis Health, Sacramento, CA 95817, USA; ftassone@health.ucdavis.edu (F.T.); rjhagerman@health.ucdavis.edu (R.J.H.); 6 Department of Biochemistry and Molecular Medicine, School of Medicine, University of California Davis Health, Sacramento, CA 95616, USA; 7 Department of Pediatrics, School of Medicine, University of California Davis Health, Sacramento, CA 95616, USA

**Keywords:** premutation, FXTAS, *FMR1* gene, episodic memory, working memory, sensorimotor function

## Abstract

Background/Objectives: Fragile X-associated tremor/ataxia syndrome (FXTAS) is characterized by tremor, gait ataxia, and cerebellar white matter degeneration, along with possible cognitive and cerebral changes. Although diagnostic criteria were originally developed in males, emerging evidence suggests that FXTAS may present differently in females. The present study examined sensorimotor and memory features of aging in female premutation carriers with (FXTAS+) and without FXTAS (FXTAS−). Methods: We studied 51 female premutation carriers (FXTAS+ = 16, FXTAS− = 35) and 24 age-matched female controls. Participants ranged in age from 47–80 years. All participants completed genetic testing, clinical evaluations, T2-weighted MRIs, and quantitative assessments of sensorimotor (precision grip force task) and memory (reading span; visual paired associates task) functions. Results: During precision grip testing, FXTAS+ carriers showed higher sustained force regularity than FXTAS− carriers (*p* = 0.03, *d* = 1.0) and controls (*p* = 0.004, *d* = 1.1) at low gain levels only. FXTAS+ participants were slower than controls on motor reaction time (*p* = 0.009, *d* = 0.82). Initial force output was higher in FXTAS+ than FXTAS− carriers (*p* = 0.03, *d* = 1.0) and controls (*p* = 0.03, *d* = 1.0) but at low gain only. FXTAS+ carriers exhibited poorer working memory than FXTAS− carriers (*p* = 0.03, *d* = 0.91) and controls (*p* = 0.02, *d* = 1.0). During a long-term memory task, FXTAS+ participants were less accurate than FXTAS− carriers (*p* = 0.04, *d* = 0.86) and controls (*p* = 0.004, *d* = 1.1) and showed increased reaction times relative to FXTAS− carriers (*p* = 0.03, *d* = −0.82) and controls (*p* = 0.01, *d* = −1.2). Conclusions: Together, these findings indicate that FXTAS+ females exhibit distinct motor and cognitive impairments, underscoring the value of quantitative behavioral measures for detecting and tracking neurodegenerative progression in female premutation carriers.

## 1. Introduction

Fragile X-associated tremor/ataxia syndrome (FXTAS) is a neurodegenerative disorder impacting a subset of individuals with the premutation allele of the Fragile X Messenger Ribonucleoprotein 1 (*FMR1*) gene located on the X chromosome [1,2]. The premutation is defined by the presence of 55–200 CGG repeats and is characterized by elevated *FMR1* mRNA expression, leading to RNA toxicity. The molecular mechanisms contributing to FXTAS, however, are not fully understood [3,4]. Primary symptoms include intention tremor and gait ataxia [1,2,5], as well as degeneration of the middle cerebellar peduncle and brainstem [6]. Additional clinical features may include executive dysfunction and memory impairments [7,8,9]. Brain changes, including white matter disease of the splenium of the corpus callosum, generalized cerebral atrophy, and cortical white matter disease, can also be evident [10,11].

FXTAS typically emerges in the early to mid-60s, and its penetrance increases with age; however, most research to date has been based on male samples. For example, 40–50% of male carriers will develop FXTAS by age 60, but up to 75% will develop FXTAS by age 80 [12]. Female premutation carriers appear to be less likely to develop FXTAS; current estimates range between 16 and 20% [13]. Few studies have rigorously examined FXTAS prevalence in female premutation carriers using gold-standard diagnostic approaches (i.e., clinical exam and T2-weighted MRI), and no known longitudinal or meta-analytic studies have examined FXTAS penetrance in females over time. These studies are needed to better understand FXTAS likelihood, presentation, and course in aging female premutation carriers. Despite this, more recent evidence suggests that the proportion of female premutation carriers impacted by FXTAS may be higher than originally estimated [6]. Knowledge of the true incidence of FXTAS in females is limited because FXTAS in females has been understudied.

FXTAS diagnostic criteria were originally developed based on studies of male carriers [2], and it remains unclear whether the disorder manifests in the same way in females. Emerging work suggests that FXTAS may present differently in females, potentially in the severity and age of onset of motor and cognitive symptoms. Previous research has suggested that progression of ataxia and tremor is less rapid in females compared to males and that females may experience psychiatric and cognitive changes during aging, though it is not yet clear whether these are more common in FXTAS or represent distinct processes [14,15]. Neuroimaging studies have also identified sex-related differences in white matter disease, as males more frequently show middle cerebellar peduncle (MCP) hyperintensities on T2-weighted MRI, whereas females rarely show these MCP signs and more frequently show involvement of the splenium of the corpus callosum [15,16]. Based on this evidence, we hypothesize that FXTAS+ females may show separate patterns of motor and cognitive deficits and decline relative to what has been documented in FXTAS+ males.

Although the characterization of FXTAS in females remains limited, recent work has started to identify specific motor and cognitive changes associated with aging in female premutation carriers. Specifically, these studies observed that female premutation carriers have demonstrated multiple motor changes relative to age-matched females without premutation alleles, including reduced fine and gross motor speed [15,17,18]. These studies have relied on standardized tests of motor function to show that motor deficits are more severe in female premutation carriers with FXTAS (FXTAS+) relative to female premutation carriers without FXTAS (FXTAS−). Still, the underlying motor control processes remain poorly understood. This gap limits progress in determining underlying mechanisms and establishing quantitative biomarkers capable of reliably differentiating FXTAS+ and FXTAS− females.

Several quantitative studies of motor behavior have offered promise as diagnostic biomarkers capable of differentiating FXTAS+ and FXTAS− females. Studying a small sample of aging female premutation carriers (ages 46–77 years), Wang et al. [19] documented reduced postural stability relative to age-matched controls across multiple standing conditions. Relative to age-matched controls, all premutation carriers showed greater variability in their center of pressure (COP) during naturalistic standing. During dynamic conditions, in which individuals were instructed to sway as far as possible without losing balance in the anterior–posterior or mediolateral plane, reductions in sway were documented specifically in FXTAS+ females, relative to controls and FXTAS− females (*n* = 5). Findings of increased COP variability suggest a reduced ability to integrate multisensory feedback to maintain stability during quiet standing. This reduced feedback control appears to contribute to reduced stability during more dynamic conditions in which posture is intentionally shifted. Consistent with these findings, our group has documented that increased variability in precision grip force is highly associated with more severe clinically rated neuromotor issues among aging female premutation carriers, though this study did not include a sufficient number of FXTAS+ females to differentiate subgroups [20]. Importantly, premutation carriers also showed lower entropy during sustained grip force, suggesting a reduced ability to integrate multiple processes to support fine motor precision that occur along different time scales. Reaction time was also elevated, implicating a delayed speed of processing or an increase in the ability to rapidly execute basic motor commands.

Aging female premutation carriers exhibit reduced cognitive abilities compared to age-matched females without premutation alleles. Prior work has reported deficits in working memory [21,22], but see Schneider et al. [15] for contrasting findings, and in long-delay verbal recall [23]. However, memory functioning in FXTAS+ females remains poorly understood because few studies have directly compared FXTAS+ with FXTAS− carriers. Existing comparisons of memory abilities in FXTAS+ and FXTAS− females have relied on brief cognitive subtests embedded in broader neuropsychological batteries (e.g., Wechsler Adult Intelligence Scale) and have typically included male and female participants. The present study advances this literature by assessing memory in greater depth, using laboratory-based measures of both verbal working and visual long-term memory. Additionally, unlike most prior FXTAS studies, which have focused on male premutation carriers, the present study specifically examines aging female carriers, including both FXTAS+ and FXTAS− participants.

The goal of the present study was to identify quantitative differences in discrete motor and memory abilities that may be useful for differentiating FXTAS+ and FXTAS− female premutation carriers. We examined multiple motor control processes, including both initial action output guided by predictive and feedforward control processes, as well as reactive motor adjustments made during sustained actions guided by sensory feedback control processes. Based on prior studies, we predicted that, relative to FXTAS− carriers and controls, FXTAS+ carriers would exhibit impaired motor performance characterized by (1) motor slowing as indicated by increased reaction time (RT) and reduced rate of initial increases in force output, and (2) atypical sensory feedback control of motor behavior as indicated by increased variability during sustained gripping. Both episodic long-term memory and working memory were also examined. We predicted that FXTAS+ carriers would show slower reaction times and reduced accuracy on both episodic long-term memory and working memory tasks compared to FXTAS− carriers and controls. Finally, we examined relationships between behavioral performance and clinical and genetic outcomes. Specifically, we expected that more severe motor and cognitive impairments would be associated with more severe clinically rated FXTAS symptoms and increased CGG repeat length after accounting for variable activation ratios (proportion of cells expressing the normal allele on the active X chromosome) across females.

## 2. Materials and Methods

### 2.1. Participants

Fifty-one *FMR1* premutation carriers, including 35 FXTAS− females and 16 FXTAS+ females, and 24 female controls completed tests of precision grip force control, episodic long-term and working memory, standardized cognitive and clinical testing, and T2-weighted MRI for diagnostic determination (see below; Table 1). Controls were matched on age with FXTAS+ carriers, and both groups were older than the FXTAS− group.

Premutation carriers were recruited from local Fragile X groups and from mailings through the National Fragile X Foundation (NFXF), as well as the Fragile X Syndrome Research and Treatment Foundation (FRAXA), the International *FMR1* premutation registry [24], and local research registries at the University of Kansas. Control participants were recruited through community advertisements, partnerships with local community groups, and our research registries.

Premutation carriers completed a clinical neuromotor evaluation, including the Fragile X-Associated Tremor and Ataxia Syndrome Rating Scale (FXTAS-RS), administered and rated by certified neurologists with established reliability with the test developers. The FXTAS-RS is a neurological evaluation of tremor, gait, and ataxia symptoms while completing various tasks such as pouring water, tandem walking, and handwriting. Higher scores reflect more pronounced physical symptoms. FXTAS diagnostic determination was based on analysis of the clinical evaluation and T2-weighted MRI using established diagnostic criteria [2]. Multiple clinical rating scales were also administered to assess psychiatric and cognitive features implicated in *FMR1* premutation carriers, including the Beck Depression Inventory (BDI), Beck Anxiety Inventory (BAI), California Verbal Learning Test 3 (CVLT), Wechsler Abbreviated Scale of Intelligence 2 (WASI), and Behavioral Dyscontrol Scale of executive abilities 2 (BDS). Results of clinical, radiological, and cognitive assessments were discussed for each premutation carrier with our clinical research team so that diagnostic determinations could be made. FXTAS+ participants met criteria for Possible (*n* = 7), Probable (*n* = 7), or Definite FXTAS (*n* = 2).

Participants were excluded for history of substance abuse or dependence within the last 6 months. Additional exclusion criteria for controls were as follows: 1) having a first- or second-degree relative with diagnosed or suspected autism spectrum disorder, intellectual disability, speech/language impairment, Fragile X syndrome, ADHD/ADD, or other neurodevelopmental condition; 2) having a personal history of a significant psychiatric disorder (which may include but is not limited to Bipolar Disorder, Schizophrenia, or any Axis II psychiatric condition) or neurologic disorder; or 3) currently taking any medication that interferes with the laboratory procedures.

The study protocol was approved by the Institutional Review Board (IRB) at the University of Kansas Medical Center, and informed consent was provided by all participants. Participants received monetary compensation for their time, and travel expenses were reimbursed if they traveled more than 200 miles round-trip.

### 2.2. Procedure

Participants completed behavioral, clinical, and MRI testing across two study sessions scheduled to minimize fatigue. During the first day of testing, participants completed tests of cognitive and motor functions, as well as clinical self-report measures of psychiatric function. During the second day of testing, clinical diagnostic and MRI testing, along with the blood draw, were completed. During MRI testing, functional MRI and separate T1-weighted and diffusion tensor imaging (DTI) sequences were collected as part of a larger study of brain changes in FXTAS, though we only report diagnostic results from analysis of T2-weighted scans here.

### 2.3. Molecular Testing

Peripheral blood samples were collected from all participants. Genomic DNA was isolated from 3 mL of whole blood using a standard procedure (Qiagen, Valencia, CA, USA). *FMR1* CGG repeat allele size was assessed by PCR and Southern blot analysis as previously described [25,26]. Activation ratio was measured by Southern blot analysis as reported in Tassone et al. [27]. The AR value indicates the percentage of cells carrying the normal allele on the active X chromosome. Allele size was averaged for cases presenting with two alleles of different repeat lengths.

### 2.4. Motor Testing

**Figure 1:**
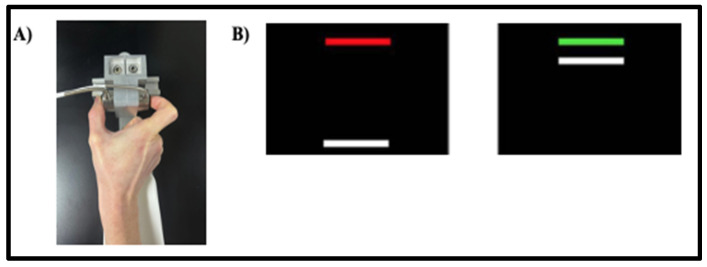
(**A**) The custom-made arm brace and load cells for precision grip testing. Participants pressed with their thumb and forefinger against two precision load cells while viewing two horizontal bars displayed vertically on the screen. (**B**) Sensorimotor test stimuli. Participants pressed when the red bar turned green to move the white bar up to the target green bar. They were instructed to maintain their force level at the level of the green bar as steadily as possible.

Tests of precision gripping were completed in a darkened room while participants were seated 52 cm from a 67 cm (27 in) Samsung LCD display monitor with a resolution of 1920 × 1080 and a 120 Hz refresh rate. They sat with their elbow positioned at 90 degrees and forearm in a secured, custom arm brace to provide stability and eliminate movement during testing (Figure 1A). Using the thumb and index finger of their dominant hand, participants pressed against the two opposing precision load cells (Model 53, Honeywell International, Inc., Columbus, OH, USA), 1.5 cm in diameter, that were secured to a custom grip device attached to the arm brace (Figure 1A). A Coulbourn (V72-25) resistive bridge strain amplifier (Coulbourn Instruments, LLC, Allentown, PA, USA) received analog signals from the load cells. Data were sampled at 100 Hz with a 16-bit analog-to-digital converter (NI USB-6341; National Instruments Corporation, Austin, TX, USA) and converted to Newtons of force using a calibration factor derived from known weights.

Participants were first assessed for maximum grip strength (maximum voluntary contraction; MVC) with their dominant hand prior to the precision grip task. They completed three trials in which they were instructed to press as hard as possible for 3 s. Maximum force output was calculated as the average of the three trials and used as the participant’s MVC. To account for individual differences in strength and ensure comparable relative exertion across participants during precision-grip testing, the target force was set to a fixed percentage of each participant’s MVC.

For the precision gripping task, participants viewed a moving white FORCE bar and a static TARGET bar that was red during rest periods and turned green during trials (Figure 1B). The FORCE bar moved upwards with increased grip force. Participants were instructed to press against the load cells as soon as the TARGET turned green and maintain their force so that the FORCE bar reached and stayed as steadily as possible at the level of the TARGET bar. Participants completed two blocks of five trials at three different visual feedback gains using their dominant hand (10 trials × 3 gain levels = 30 trials). Gain levels were varied by changing the visual angle across three levels: low (0.059°), medium (0.623°), and high (6.575°). For conditions with higher visual angles, the FORCE bar moved a greater distance per Newton change in force. Visual angles selected based on prior studies showing that force variability decreases with increased visual angle up to 1 deg [28]. Each trial lasted 15 s, followed by 15 s rest, with blocks separated by 30 s rest intervals. The order of visual gain levels was randomized across participants.

#### Force Data Processing

Procedures for processing and analyzing force data were similar to those previously reported. Force data were low-pass filtered with a doublepass 4th-order Butterworth filter at a 15 Hz cutoff and analyzed using a custom algorithm previously developed by our lab and implemented in MATLAB (version 2024b). [29].

During the initial rise phase in which individuals pressed on the load cells to reach the target level, we examined reaction time and peak rate of force increase (i.e., the maximum value of the first derivative of the force trace). The onset of the rise phase was calculated as the time at which the rate of force increase first exceeded 5% of the peak rate of force increase and remained above this level for at least 100 ms [29,30]. RT was calculated as the difference between the rise phase onset and the appearance of the start cue. The rise phase offset was calculated as the time-point when the rate of force increase fell below 5% of the peak rate of force increase, and the force level was within 90–110% of the mean force of the sustained phase [29]. The peak rate of force increase was defined as the maximum value of the first derivative of the force trace. To determine the extent to which participants could maintain a constant level of force using visual feedback, the sustained phase was examined. The sustained phase consisted of the last 8–12 s of the trial during which the participant was attempting to maintain a stable force at the level of the target. This segment was adjusted to avoid instances when the participant let go during the trial or the rise phase offset was after the first three seconds of the trial (e.g., the maximum 12 s segment of ‘sustained’ phase data could not be achieved). The variability of the force time series was calculated using the following procedures: First, force data were linearly detrended to account for systematic changes in mean force over the course of the trial (e.g., data drift). Second, the within-trial standard deviation (SD) of the force time series was calculated. Based on findings that force variability scales with the amount of force that is exerted, we examined the coefficient of variation (CoV) calculated as the SD of sustained force/mean force. To examine the time-dependent structure of the time series, the approximate entropy (ApEn) was calculated for each trial [31,32]. ApEn returns a value between 0 and 2, reflecting the predictability of future values in a time series based on previous values. For example, a sine wave has accurate short- and long-term predictability, corresponding to an ApEn value near 0. High irregularity of the data, reflective of the independence of each force value, returns an ApEn near 2. The algorithm and parameter settings for these calculations (m = 2; r = 0.2 SD of the signal) were identical to previous work [33]. Sustained phase variables were excluded if fewer than 8 s of data were available or if participants returned to baseline for more than 1 s (e.g., a > 1 s dip of the force signal).

### 2.5. Memory Testing

Memory tests were created and administered using PsychoPy3 [34] and administered on a 60.96 cm (24 in) Dell LCD display monitor with a resolution of 1920 × 1080 and a 60 Hz refresh rate. For the working memory task, participants used a custom-made button box that recorded finger presses through a USB port with a sampling rate of 125 Hz.

#### 2.5.1. Working Memory

To examine working memory, we administered a reading span task (RSPAN), in which participants switched between making judgments about sentences and remembering letters, adapted from Kane et al. [35]. During testing, a sentence was displayed for four seconds, and participants were asked to decide whether the sentence was logical or illogical. Illogical sentences (e.g., “Every now and then I catch myself swimming blankly at the wall.”) were created by replacing a single word in an otherwise coherent sentence, rendering its meaning nonsensical. Participants were instructed to select ‘yes’ by pressing the left button on the button box if they believed the sentence was logical. They were instructed to select ‘no’ by using the right button on the button box if the sentence was illogical. Following the sentence, a single letter was displayed for 1 s. After all sentence–letter pairs were presented for each trial, text boxes were displayed on the screen, and participants were asked to freely recall the letters in order of presentation. The task consisted of 15 trials with set sizes presented randomly, ranging from 3–7 sentence–letter pairs. Each set size was presented twice. Response accuracy was measured as the proportion of letters correctly recalled in order. If letters were correctly recalled but in the incorrect order, responses were scored as inaccurate. Three participants (2 controls, 1 FXTAS+) were dropped from the working memory analyses because they were given a different version of the working memory span task. One participant (FXTAS+) was not administered memory tasks due to time constraints during the study session. One participant (FXTAS−) was dropped from memory analyses because they did not understand the task instructions. Therefore, 70 participants completed the working memory task.

#### 2.5.2. Episodic Long-Term Memory

**Figure 2:**
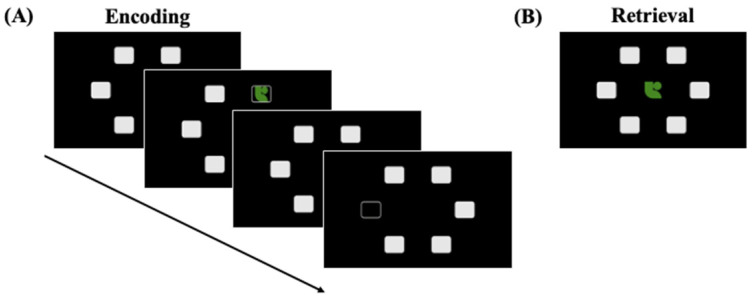
(**A**) Structure of an encoding trial for the visuospatial paired associates (visual PA) episodic long-term memory task with example stimuli. The participants were instructed to remember the location of the objects within the boxes. Participants are shown 1, 2, 3, 6, or 8 objects. (**B**) An example retrieval trial. The objects are shown in the middle of the screen, and participants are instructed to select the box in which the object appeared.

To quantify episodic long-term memory differences in premutation carriers, we administered a modified version of the CANTAB visuospatial paired associates task (visual PA, Figure 2). During the visual PA task, participants viewed six boxes arranged in a circle on the screen (Figure 2A). One, two, three, six, or eight objects were shown inside a box. Each box remained open for four seconds, followed by a one-second delay. Once all objects had been presented, each was presented in random order at the center of the screen for six seconds (Figure 2B). Participants were instructed to use the computer mouse to select the box in which the object had appeared. There were 15 total trials (two of each set size) for a total of 60 object–location pairs. Response accuracy was measured as the proportion of object–location pairs correctly recalled, and RT was recorded for correct trials. One participant (FXTAS+) was not administered memory tasks due to time constraints during the study session. One participant (FXTAS−) was dropped from memory analyses because they did not understand the task instructions. Therefore, 73 participants completed the episodic memory task.

### 2.6. Statistical Approach

Participant groups differed on chronological age (*F*(2,72) = 8.11, *p* < 0.001, partial η^2^ = 0.18), with FXTAS− carriers younger than both FXTAS+ carriers (*p* = 0.02) and controls (*p* = 0.001), while FXTAS+ and control participants did not differ on age (*p* = 0.91). Based on these findings, age was included as a covariate in all analyses to control for such differences.

Dependent measures from the precision force tests were analyzed using separate ANCOVAs, with gain level (low, medium, high) as a within-subjects factor and age as a covariate. Memory data was analyzed using three ANCOVAs, conducted separately for RSPAN performance, visual PA accuracy, and visual PA RT, controlling for age.

When omnibus tests were significant, Tukey HSD post hoc comparisons were conducted. Results of all group comparisons are reported along with any significant covariates for each model.

For the visual PA task, reaction times ±3 standard deviations from each participant’s mean (32 trials; 0.68% of the data), as well as anticipatory responses (≤150 ms; 3 trials; 0.06% of the data), were excluded from analysis.

Pearson correlation coefficients were then computed to assess relationships between memory, motor, clinical, and genetic variables.

For genetic analyses, CGG repeat values were weighted by activation ratio (AR) following the formula reported in Tassone et al. [36].

CGGobs = CGGnormal × AR + CGGpremutation × (1 − AR)(1)

## 3. Results

### 3.1. Motor Behavior

#### 3.1.1. Rapid Motor Behavior

**Figure 3:**
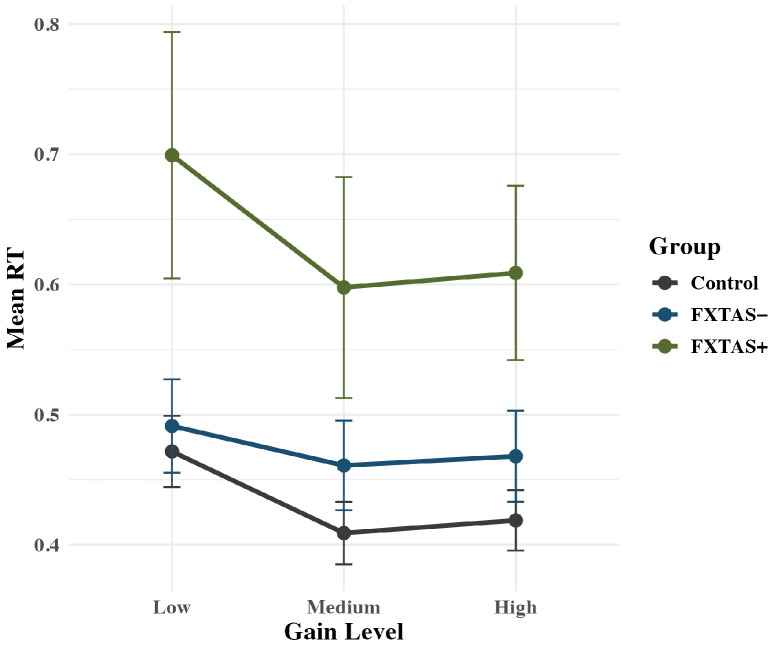
Rise latency plotted as a function of group. Raw means are plotted by group, with standard error bars. FXTAS+ participants responded significantly slower compared to FXTAS− participants and controls.

Groups differed on RT during precision gripping: *F*(2,68) = 5.89, *p* = 0.004, partial η^2^ = 0.15. FXTAS+ participants (*M* = 0.64 s; *SD* = 0.33) showed longer RTs compared to controls (*M* = 0.43 s; *SD* = 0.12; *p* = 0.009) but not FXTAS− carriers (*M* = 0.47; *SD* = 0.21; *p* = 0.07); FXTAS− carriers and controls did not differ on RT (*p* = 0.44, Figure 3).

There were no group differences in the peak rate of initial force increase: *F*(2,71) = 1.17, *p* = 0.315, partial η^2^ = 0.03. Increased age was associated with lower peak rates of force increase across gain levels: *F*(1,71) = 4.993, *p* = 0.029, partial η^2^ = 0.07. 

**Figure 4:**
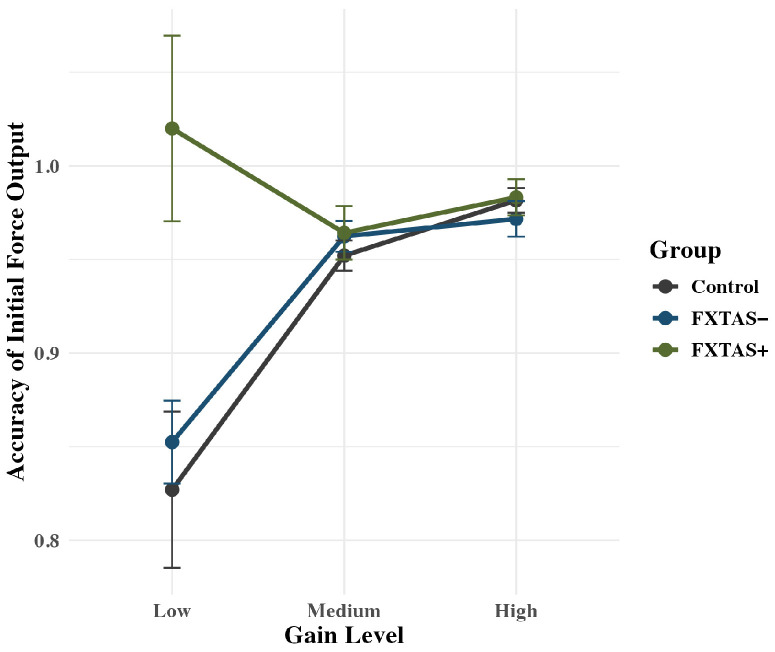
Rise accuracy is plotted as a function of group, with raw means and standard error bars. An interaction indicates that FXTAS+ participants were more accurate at the low gain condition compared to medium and high gain conditions.

The group x gain interaction term was significant for accuracy of initial force output: *F*(2,71) = 6.31, *p* < 0.001, partial η^2^ = 0.15 (Figure 4). FXTAS+ participants (*M* = 1.02; *SD* = 0.20) showed higher levels of accuracy than controls (*M* = 0.83; *SD* = 0.20; *p* = 0.03) and FXTAS− participants (*M* = 0.85; *SD* = 0.13; *p* = 0.03) at the low gain condition only. There is an overall group effect, where FXTAS+ carriers are performing more accurately regardless of gain, but the effect is being driven by the low gain level, as the other levels do not show any significant difference between groups (all *p* > 0.5).

#### 3.1.2. Sustained Motor Behavior

During sustained gripping, the group x gain interaction term was significant for sustained accuracy: *F*(2,69) = 8.34, *p* < 0.001, partial *η^2^* = 0.20. FXTAS+ participants (*M* = 1.02; *SD* = 0.17) showed higher levels of accuracy than controls (*M* = 0.84; *SD* = 0.15; *p* = 0.004) and FXTAS− participants (*M* = 0.88; *SD* = 0.10; *p* = 0.03) at the low gain condition only. There is an overall group effect, where FXTAS+ carriers are performing more accurately regardless of gain, but the effect is being driven by the low gain level, as the other levels do not show any significant difference between groups (all *p* > 0.8).

**Figure 5:**
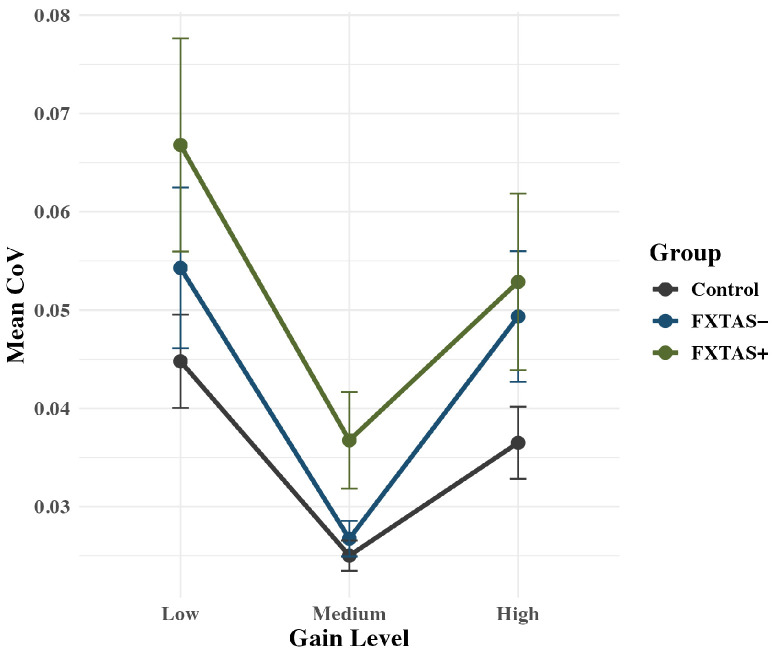
Force variability (CoV) plotted as a function of group and gain level. Raw means are plotted by group, with standard error bars. FXTAS+ carriers exhibited greater variability than controls at the medium and high force levels, whereas FXTAS− carriers differed from controls at the low and high levels. FXTAS+ and FXTAS− groups differed only at the medium force level.

Analysis of CoV showed a Group x Gain quadratic interaction effect that approached significance: *F*(2,71) = 2.90, *p* = 0.060, partial η^2^ = 0.08 (Figure 5). Based on the trend level significance, we show post hoc analyses of the interaction as well as the main group effect below. In terms of the interaction, at low gain, FXTAS+ (*M* = 0.067; *SD* = 0.04) and FXTAS− (*M* = 0.054; *SD* = 0.05) carriers showed elevated variability (CoV) compared to controls (*M* = 0.045; *SD* = 0.02), though this effect was only significant for FXTAS− carriers (*p*’s = 0.055 and 0.029, respectively). The FXTAS+ and FXTAS− carriers did not differ in variability at low gain (*p* = 0.967). At medium gain, FXTAS+ carriers (*M* = 0.037; *SD* = 0.02) showed elevated force variability compared to both FXTAS− carriers (*M* = 0.027; *SD* = 0.01; *p* = 0.051) and controls (*M* = 0.025; *SD* = 0.01; *p* = 0.003). FXTAS− carriers and controls were not different in their CoV at medium gain (*p* = 0.192). At high gain, FXTAS+ (*M* = 0.053; *SD* = 0.04) and FXTAS− carriers (*M* = 0.049; *SD* = 0.04) each showed greater force variability than controls (*M* = 0.037; *SD* = 0.02), though differences were significant only for FXTAS+ carriers (*p*’s = 0.100 and 0.034). FXTAS+ and FXTAS− carriers did not differ from one another on CoV at high gain (*p* = 0.777). The overall effect of group was significant as well: *F*(2,71) = 4.99, *p* = 0.009, partial η^2^ = 0.12, with FXTAS+ (*M* = 0.052; *SD* = 0.04) and FXTAS− participants (*M* = 0.043; *SD* = 0.04) each showing higher variability than controls (*M* = 0.035; *SD* = 0.02; p’s = 0.010 and 0.008, respectively). Variability did not differ between FXTAS+ and FXTAS− carriers (*p* = 0.837).

### 3.2. Memory Function

#### 3.2.1. Working Memory Function

**Figure 6:**
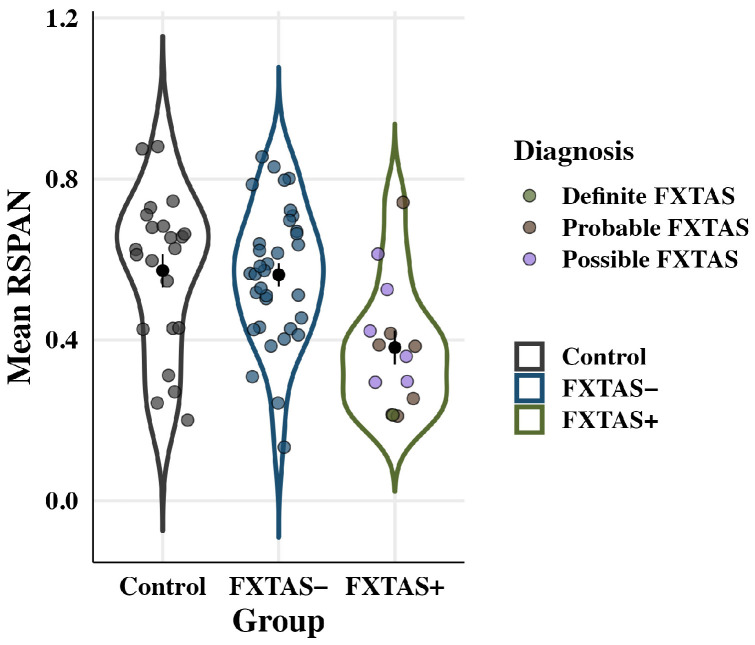
Working memory (RSPAN) accuracy by group. Colored points represent individual scores; black points show raw means with standard error bars. FXTAS+ participants recalled fewer items than FXTAS− participants and controls.

Working memory performance is plotted by group in Figure 6. Groups differed on their working memory accuracy: *F*(2,64) = 6.1, *p* = 0.004, partial η^2^ = 0.16. FXTAS+ participants (*M* = 0.38; *SD* = 0.16) showed reduced working memory accuracy compared to both FXTAS− participants (*M* = 0.56; *SD* = 0.17; *p* = 0.03, partial η^2^ = 0.10) and controls (*M* = 0.57; *SD* = 0.19; *p* = 0.02, partial η^2^ = 0.10). FXTAS− participants and controls did not differ in their working memory performance (*p* = 0.90, partial η^2^ = 0.003). There was no effect of age: *F*(1,64) = 0.43, *p* = 0.51, partial η^2^ = 0.007, and no group x age interaction: *F*(2,64) = 0.45, *p* = 0.64, partial η^2^ = 0.01.

#### 3.2.2. Episodic Long-Term Memory Function

**Figure 7:**
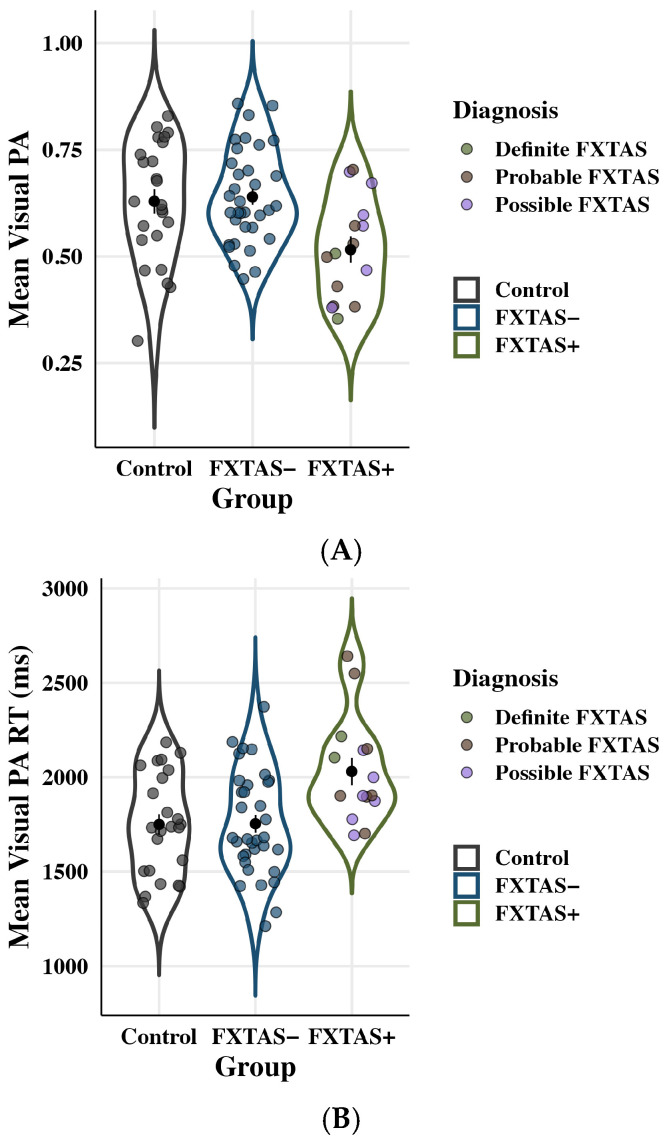
Long-term memory (Visual PA) RT outcomes. Colored points represent individual scores; black points show raw means with standard error bars. (**A**) Visual PA accuracy as a function of group. FXTAS+ participants showed decreased accuracy relative to FXTAS− participants and controls. (**B**) Visual PA RT as a function of group. FXTAS+ participants exhibited slowed reaction time compared to both FXTAS− participants and controls.

Accuracy and RT on the visual PA task are plotted by group in Figure 7. Groups differed on visual PA accuracy: *F*(2,67) = 5.97, *p* = 0.004, partial η^2^ = 0.15. FXTAS+ individuals (*M* = 0.51; *SD* = 0.12) showed reduced accuracy compared to both FXTAS− (*M* = 0.64; *SD* = 0.11; *p* = 0.03, partial η^2^ = 0.09) and control participants (*M* = 0.63; *SD* = 0.14; *p* = 0.01, partial η^2^ = 0.12). No differences between FXTAS− and controls were observed (*p* = 0.72, partial η^2^ = 0.01). Visual PA accuracy was also inversely associated with age: *F*(1,67) = 4.49, *p* = 0.04, partial η^2^ = 0.06, though age effects did not vary as a function of group membership, group x age interaction: *F*(2,67) = 0.70, *p* = 0.50, partial η^2^ = 0.02. 

RT on the visual PA test differed across groups: *F*(2,67) = 6.64, *p* = 0.002, partial η^2^ = 0.17. FXTAS+ (*M* = 2030.30 ms; *SD* = 278.89 ms) participants had significantly slower RTs than FXTAS− (*M* = 1753.42 ms; *SD* = 275.32; *p* = 0.04, partial η^2^ = 0.08) and control participants (*M* = 1749.86 ms; *SD* = 266.56 ms; *p* = 0.004, partial η^2^ = 0.14). Differences between FXTAS− and controls were not significant (*p* = 0.42, partial η^2^ = 0.02). RT also increased with age: *F*(1,67) = 7.32, *p* = 0.01, partial η^2^ = 0.10, though the group x age interaction was not significant: *F*(2,67) = 0.41, *p* = 0.67 partial η^2^ = 0.01.

### 3.3. Correlations Between Motor and Memory Measures

Memory measures were not significantly correlated with motor variables in any group except the FXTAS− group, where accuracy on the visual paired-associates (PA) task was positively correlated with average peak velocity (Table A1). Although other correlations did not reach statistical significance, several weak to moderate associations emerged in the data. Notably, reaction time on the visual PA task in the FXTAS+ group showed a moderate positive relationship (r = 0.39) with average latency on the motor task.

### 3.4. Correlations Between Motor, Genetic, and Clinical Measures

Full correlation matrices with all variables and all groups are available in the Appendix A (Table A2). As expected, the relationships amongst the motor measures were significantly correlated within each of the groups (Table A2). These motor measures were moderately associated with the clinical outcome measures within the control group (e.g., with CVLT performance; see Table A2); however, they were weak or absent within both carrier groups (Table 2). Correlations within the carrier groups are shown in Table 2. Genetic measures showed modest but consistent relationships with motor performance, particularly in carriers. In FXTAS− females, higher CGG repeat length and lower activation ratio tended to relate to greater motor variability and reduced accuracy. Among FXTAS+ carriers, these associations appeared stronger, with CGG metrics (RAW CGG, CGGobs, and Activation Ratio) related to greater motor irregularity and reduced control precision.

### 3.5. Correlations Between Memory, Genetic, and Clinical Measures

Full correlation matrices with all variables and all groups are available in the Appendix A (Table A3). Across premutation groups, laboratory-based memory measures were moderately intercorrelated (Table 3), whereas these relationships were weaker among controls (Table A3). Performance on the lab-based memory tasks was also moderately related to standardized verbal memory performance on the CVLT measures. Within the FXTAS+ group, several moderate, negative associations emerged between clinical severity (FXTAS-RS) and memory performance, such that higher FXTAS-RS scores were associated with poorer visual paired associates accuracy (*r* = −0.40) and reduced working memory accuracy on the RSPAN (*r* =−0.36; Table 3).

## 4. Discussion

This is the first known study to examine multiple quantitative motor and cognitive traits in female premutation carriers with and without FXTAS. Our main goal was to identify differences in discrete motor and memory abilities that may be useful for differentiating FXTAS+ and FXTAS− female premutation carriers, and the current results supported several of our hypotheses. 

### 4.1. Reaction Times

We found that, as predicted, female premutation carriers with FXTAS showed increased RT across motor and memory processes relative to both FXTAS− carriers and controls. These findings suggest that disease-specific processes slow either processing or movement speed, or both, in female premutation carriers. The relationship between RT during precision gripping and memory responding was modest but significant across the full sample, and the relationship was moderate but not significant when analyzing the FXTAS+ subgroup alone. These results suggest that multiple overlapping processes may affect the speed with which females can carry out motor and memory tasks. For example, it is possible that white matter degeneration may disrupt both movement speed and speed of information processing, but the extent to which these separate abilities are impacted in different individuals may vary as a function of localization or disease stage. This hypothesis is consistent with the well-documented white matter degeneration in males and females with FXTAS [6,37,38], including quantitative studies of white matter microstructural integrity [39,40]. These findings are also consistent with studies demonstrating that parkinsonian motor symptoms, including bradykinesia, are common in FXTAS, and suggest that tracking movement and cognitive slowing may be important for tracking FXTAS onset or progression in female premutation carriers [1,2]. Studies examining changes in motor and memory slowing in relation to neuroanatomical changes in white matter pathways will be important for clarifying the extent to which these markers of FXTAS degeneration in females may reflect overlapping or distinct mechanisms.

### 4.2. Initial Force Output

**Figure A1:**
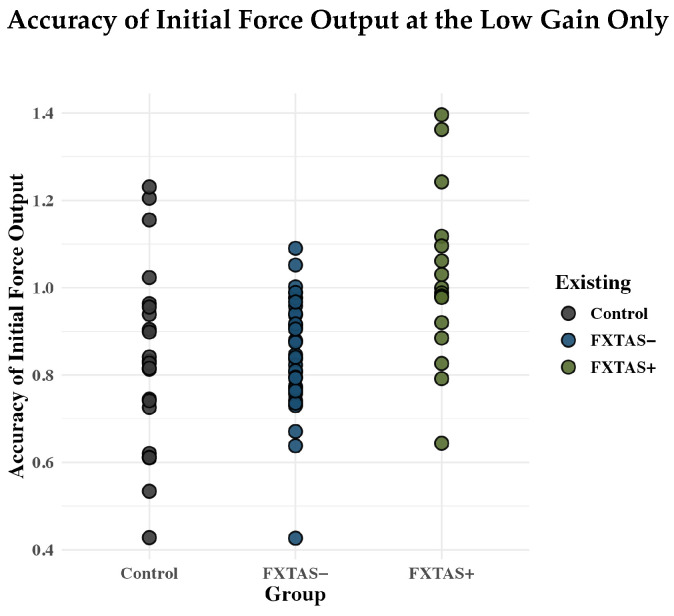
Raw values of rise accuracy are plotted for each participant in the low gain level only. FXTAS+ females showed increased force relative to FXTAS− carriers and controls.

Contrary to our predictions, we did not observe group differences in the rate of initial force output. However, during initial gripping, FXTAS+ females did show increased force relative to FXTAS− carriers and controls at the lowest gain. These findings reflect a higher proportion of FXTAS+ carriers relative to FXTAS− carriers and controls who overshot the force target with their initial output (see Figure A1). During the initial phase of precision gripping, in which individuals rapidly increase their force output, accuracy is primarily supported by action plans that are subsequently modified by sensory feedback available on a more protracted time course. To optimize motor output, individuals may undershoot targets before sensory feedback can be integrated to guide more precise behavior [29]. This approach is more efficient than overshooting targets and then having to decrease force output to “reverse course”. Our finding that FXTAS+ females more frequently overshoot the target during initial action output implicates feedforward mechanisms involved in guiding behavior prior to slower sensory feedback processes being available to support reactive motor adjustments.

### 4.3. Sustained Force Output

As predicted, FXTAS+ premutation carriers exhibited increased variability during precision gripping relative to FXTAS− carriers and controls. Increased motor variability during precision gripping for FXTAS+ and FXTAS− premutation carriers suggests that sensory feedback modulation of motor output is impaired. These results are consistent with prior studies showing increased variability during postural control [19] and during precision gripping in premutation carriers that scales with clinically rated FXTAS severity [20,41]. Our findings extend these prior studies by demonstrating that increased variability during precision gripping may be more severe in FXTAS+ females compared to FXTAS− females at select gain levels. Elevations in variability for FXTAS+ carriers compared to FXTAS− carriers at medium gain were only significant at a trend level (*p* = 0.061), suggesting that this effect is modest, and/or our ability to detect a significant difference was limited by the relatively small sample of FXTAS+ females studied here. It is important to note that we saw larger ranges of variability within both our FXTAS+ and FXTAS− carriers relative to controls, indicating greater heterogeneity in sensory feedback control of motor behavior. These findings indicate that premutation carriers are variably affected, and that deterioration of sensory feedback control processes may scale with FXTAS progression, as suggested by the moderate (though non-significant) associations with clinically rated FXTAS symptoms (r’s = 0.029 and 0.031, respectively). Overall, findings of increased variability in female premutation carriers implicate cerebellar mechanisms that modulate cortical motor output via sensory feedback error. We previously documented that increases in grip force variability were associated with reduced extrastriate–cerebellar Crus I functional connectivity, implicating both key visuomotor networks and white matter pathways involving posterior tracts [41]. These prior functional neuroimaging results implicate white matter tracts involving the splenium of the corpus callosum, consistent with studies showing that splenium degeneration may be the most common neuropathological finding in FXTAS+ females [42]. It will be important to examine motor variability in aging FXTAS+ and FXTAS− female carriers to determine the extent to which sensory feedback motor traits and their functional and structural brain substrates may show changes that track with FXTAS risk or progression.

### 4.4. Working and Long-Term Memory

In line with our hypotheses, FXTAS+ female premutation carriers exhibit reduced working memory and long-term memory relative to FXTAS− carriers and controls, while FXTAS− carriers do not differ from controls. It is worth noting that FXTAS+ carriers showed both lower accuracy and slower RTs on these tasks compared to the other groups, indicating that performance differences were not due to a speed–accuracy trade-off. Working memory deficits observed in FXTAS+ females may reflect disruptions in white matter integrity, particularly within frontoparietal white matter tracts [37] that support communication between prefrontal and parietal cortices and are particularly important for working memory [43]. Damage to these pathways could impair the integration of sensory feedback and executive control processes that are essential for maintaining and manipulating information in working memory. These findings are consistent with prior studies reporting executive function deficits in female premutation carriers, particularly those with FXTAS, as well as in men with FXTAS [44].

Similarly, long-term memory deficits in FXTAS+ carriers may stem from structural and functional changes in the hippocampus and parahippocampal regions, coupled with widespread white matter loss. These changes may degrade the quality of stored memory representations and contribute to slower response times during retrieval. Thus, quantitative reductions in executive and memory abilities may serve as important markers of FXTAS-related neurodegeneration in female premutation carriers, as evidenced by FXTAS+ specific differences on working and episodic memory tasks.

Notably, the observed correlation between working memory and episodic memory performance suggests that these domains share overlapping mechanisms. This overlap likely reflects impairments in processes that support the encoding, maintenance, and retrieval of new information over short and long delays. Together, these findings indicate that disruptions in both hippocampal–medial temporal circuits and frontoparietal white matter pathways may jointly contribute to the broader memory impairments seen in FXTAS+ female carriers.

By contrast, FXTAS− participants did not differ from controls on any memory measures (working or long-term memory accuracy, or long-term memory RT), suggesting that cognitive differences in aging female premutation carriers may be specific to those who develop FXTAS. This distinction underscores the need for longitudinal studies to clarify age-related trajectories and identify biomarkers predictive of cognitive or motor changes in asymptomatic female premutation carriers.

### 4.5. Clinical and Genetic Correlations

As predicted, we saw modest associations among premutation carriers (both FXTAS+ and FXTAS−) between several quantitative motor and memory abilities with both CGG repeat length and clinically rated FXTAS severity. Associations with CGG repeat length were stronger when accounting for activation ratios. Many of these correlations were not significant, but they are interpreted here because of their medium effect sizes in the context of a relatively small sample of FXTAS+ carriers, and to highlight several important considerations. First, associations between quantitative motor and memory traits with CGG repeat length were stronger when accounting for activation ratios. Analysis of associations between CGG repeat expansion size and clinical outcomes has leveraged multiple strategies to account for (or not account for) variable expression across cells of the premutation allele, and these results suggest that previously proposed formulas for weighting repeat length by AR will be important for understanding mechanistic linkages with different clinical traits or biomarkers. Second, we found that motor, working memory, and episodic memory abilities each showed relationships with clinically rated FXTAS symptom severity, suggesting that cognitive changes may track with motor deterioration in females with FXTAS. Quantitative monitoring of motor and cognitive abilities, including both working and episodic memory abilities, may be important for identifying FXTAS in females and for understanding basic mechanisms of neurodegeneration that appear to differ across sexes [15,16].

One final important finding from our study to highlight is that out of the 51 women we recruited, 16 were ultimately diagnosed with FXTAS (31% were FXTAS+). These diagnoses were made after comprehensive clinical evaluations, MRI assessments, and expert consensus among specialists in FXTAS and movement disorders. This is notable because it suggests that the proportion of female premutation carriers who develop FXTAS during middle to later adulthood may be higher than what earlier reports indicated. However, given the modest sample size, replication in larger, independent cohorts is needed to refine estimates of FXTAS prevalence among female premutation carriers.

### 4.6. Limitations

While we present multiple novel findings of quantitative deficits in motor and cognitive functions in FXTAS+ females, these results should be interpreted in the context of several limitations. First, age is a critical factor in the emergence and severity of FXTAS, and in the current study, the FXTAS− group was significantly younger than both the FXTAS+ and control groups. While we controlled for age differences in our analyses, the cross-sectional design and lack of age-matching across groups limit inferences about the timing and progression of deficits. Strict age-matched cross-sectional and longitudinal studies will be critical for determining whether our measures or a combination of our measures and clinical/MRI tests can predict disease onset or course. We are currently tracking participants in this study over time so that we may determine whether the quantitative measures reported here track with disease progression and/or are predictive of disease onset or progression. Second, we had an unbalanced design: the FXTAS+ group was smaller than the FXTAS− and control groups. However, this imbalance reflects the prevalence rates of FXTAS amongst females. Future research should aim for larger and more balanced group sizes to enhance statistical power and the reliability of group comparisons. Third, the inclusion of a direct measure of processing speed in future studies would help determine whether the prolonged response times observed in FXTAS+ participants primarily reflect slowed cognitive processing, motor execution, or a combination of both. This would provide greater specificity regarding the nature of the deficits contributing to the delayed performance across motor and memory domains in the FXTAS+ group. Lastly, current diagnostic criteria for FXTAS were formulated largely on males, and several studies have suggested that both behavioral and brain features of FXTAS may be different across males and females. Hall et al. [6] proposed that different neuroanatomical indicators of FXTAS should be considered, though direct comparisons of quantitative behavioral and brain features of FXTAS across males and females are critically needed to clarify whether distinct symptom features should be established.

## 5. Conclusions

Overall, our findings demonstrate that FXTAS+ females show multiple quantitative deficits in motor and cognitive processes compared to asymptomatic premutation carriers. We document both deficient feedforward and sensory feedback processes that are specific to FXTAS+ female premutation carriers. Similarly, response times and accuracy on working and episodic memory are selectively impacted in FXTAS+ females compared to asymptomatic female premutation carriers, suggesting that cognitive processes that are not considered core to FXTAS may be important to track for understanding degeneration in females.

## Figures and Tables

**Table 1:** Demographic, cognitive, and clinical characteristics (mean ± SD).

Variable	Controls (*n* = 24)	FXTAS− (*n* = 35)	FXTAS+ (*n* = 16)	*p*
Age (yrs)	66.6 ± 6.3	59.3 ± 8.3	65.7 ± 7.1	<0.001 *
Education (yrs)	17.8 ± 1.9	16.8 ± 2.9	16.3 ± 2.3	0.16 *
CGG Repeat Length	----	88.3 ± 14.6	95.1 ± 20.5	0.15 #
CGG Repeat Length Range	----	61–114	61–145	----
Activation Ratio	----	0.6 ± 0.2	0.5 ± 0.2	0.10 #
FXTAS-RS	----	3.6 ± 3.3	12.4 ± 7.2	**<0.001 #**
FSIQ	119.3 ± 9.8	114.2 ± 10.2	113.9 ± 12.6	0.16 *
SDFR	12.5 ± 2.5	12.5 ± 3.2	11.0 ± 3.1	0.20 *
SDCR	12.2 ± 3.2	12.3 ± 3.7	11.1 ± 2.5	0.46 *
LDFR	12.7 ± 2.2	12.5 ± 3.2	10.0 ± 2.6	0.007 *
LDCR	12.1 ± 2.5	12.3 ± 3.1	10.8 ± 2.9	0.21 *
BDS2	22.1 ± 1.6	22.1 ± 2.8	20.0 ± 3.2	0.02 *
BDI	3.1 ± 2.4	7.9 ± 6.7	12.9 ± 11.3	<0.001 *
BAI	1.7 ± 1.9	6.5 ± 5.6	10.3 ± 7.2	<0.001 *

* = ANOVA results for all three groups, # = t-test results for FXTAS− vs. FXTAS+ groups; significant findings in bold. Abbreviations: CGG, cytosine-guanine-guanine; FXTAS-RS, FXTAS Rating Scale; Wechsler Abbreviated Scale of Intelligence subtests: FSIQ, full-scale IQ; California Verbal Learning Task subscores: SDFR, short delay free recall; SDCR, short delay cued recall; LDFR, long delay free recall; LDCR, long delay cued recall; BDS-II, Behavioral Dyscontrol Scale; BDI, Beck Depression Inventory; BAI: Beck Anxiety Inventory.

**Table 2:** Correlations between motor, clinical, and genetic variables for premutation carriers (FXTAS+ and FXTAS−).

FXTAS+	Average Latency (Rise)	Average Peak Velocity	Accuracy of Initial Force Output (Low Gain)	Sustained Accuracy	CoV (Med Gain)	ApEn
FSIQ	−0.09	−0.36	−0.24	−0.01	0.26	−0.19
BDS	0.61 *	−0.41	−0.36	−0.30	0.04	0.32
BDI	−0.13	0.02	0.09	0.05	−0.43	0.20
BAI	−0.29	0.23	0.02	−0.03	−0.05	−0.27
LDFR	0.09	−0.02	−0.34	−0.29	−0.40	0.19
LDCR	0.06	−0.05	−0.16	−0.02	−0.45	0.21
SDFR	0.25	0.11	−0.41	−0.44	−0.10	−0.02
SDCR	0.32	−0.20	−0.33	−0.35	−0.32	0.29
Raw CGG	−0.32	0.39	0.18	0.33	−0.08	0.01
Activation Ratio	0.31	−0.26	−0.09	−0.10	0.13	−0.34
FXTAS-RS	−0.10	0.46	0.49	0.31	0.33	−0.04
CGGobs	−0.38	0.45	0.32	0.41	−0.20	0.34
FXTAS−	Average latency (rise)	Average peak velocity	Accuracy of Initial Force Output (low gain)	Sustained Accuracy	CoV (med gain)	ApEn
FSIQ	−0.15	0.30	0.26	0.15	−0.14	−0.12
BDS	0.05	−0.01	0.07	−0.11	−0.06	−0.01
BDI	0.04	0.03	0.16	0.11	0.0	0.11
BAI	−0.23	0.01	−0.04	−0.19	−0.09	0.05
LDFR	−0.12	0.33	0.34 *	0.33	−0.27	−0.09
LDCR	−0.07	0.18	0.37 *	0.24	−0.08	−0.13
SDFR	−0.19	0.26	0.32	0.23	−0.22	−0.18
SDCR	−0.06	0.23	0.39 *	0.25	−0.11	−0.17
Raw CGG	−0.04	0.14	−0.07	0.16	−0.37 *	0.0
Activation Ratio	−0.15	0.05	0.19	0.30	−0.15	−0.22
FXTAS-RS	0.03	0.04	0.05	0.19	0.29	−0.05
CGGobs	0.08	−0.02	−0.22	−0.19	−0.07	0.22

Values are Pearson correlations. Abbreviations: CGG, cytosine-guanine-guanine; FXTAS-RS, FXTAS Rating Scale; Wechsler Abbreviated Scale of Intelligence subtests: FSIQ, full-scale IQ; California Verbal Learning Task subscores: SD FR, short delay free recall; SD CR, short delay cued recall; LD FR, long delay free recall; LD CR, long delay cued recall; BDS, Behavioral Dyscontrol Scale; BDI, Beck Depression Inventory; BAI: Beck Anxiety Inventory; CoV: Force variability; ApEn: approximate entropy; *p* < 0.05 (*).

**Table 3:** Correlations between memory, clinical, and genetic variables for premutation carriers (FXTAS+ and FXTAS−).

FXTAS+	Visual PA	Visual PA RT	RSPAN
FSIQ	0.21	0.00	−0.16
BDS	0.52 *	0.35	0.15
BDI	0.05	−0.30	−0.17
BAI	−0.17	−0.34	−0.14
LDFR	0.35	−0.06	0.21
LDCR	0.40	−0.06	0.20
SDFR	0.29	−0.03	0.31
SDCR	0.54 *	0.02	0.20
Raw CGG	−0.05	−0.48	0.23
Activation Ratio	0.06	0.22	0.21
FXTAS-RS	−0.40	0.02	−0.36
CGGobs	−0.25	−0.25	−0.13
FXTAS−	Visual PA	Visual PA RT	RSPAN
FSIQ	0.42 *	0.09	0.20
BDS	0.34	0.00	0.18
BDI	0.26	−0.06	−0.07
BAI	0.05	0.19	−0.06
LDFR	0.20	0.22	0.20
LDCR	0.15	0.25	0.24
SDFR	0.17	0.20	0.19
SDCR	0.17	0.30	0.20
Raw CGG	0.09	−0.15	0.11
Activation Ratio	−0.20	0.04	0.25
FXTAS-RS	−0.05	−0.01	−0.05
CGGobs	0.22	−0.19	−0.16

Values are Pearson correlations. Abbreviations: CGG, cytosine-guanine-guanine; FXTAS-RS, FXTAS Rating Scale; Wechsler Abbreviated Scale of Intelligence subtests: FSIQ, full-scale IQ; California Verbal Learning Task subscores: SD FR, short delay free recall; SD CR, short delay cued recall; LD FR, long delay free recall; LD CR, long delay cued recall; BDS, Behavioral Dyscontrol Scale; BDI, Beck Depression Inventory; BAI: Beck Anxiety Inventory; Visual PA: visual paired associates accuracy; Visual PA RT: visual paired associates reaction time; RSPAN: reading span accuracy; *p* < 0.05 (*).

## Data Availability

The raw data supporting the conclusions of this article will be made available by the authors on request.

## Abbreviations

The following abbreviations are used in this manuscript:

*FMR1*	Fragile X Messenger Ribonucleoprotein 1
FXTAS	Fragile X-associated tremor/ataxia syndrome
FXTAS+	Premutation carrier with FXTAS
FXTAS−	Premutation carrier without FXTAS
COP	Center of pressure
RT	Reaction Time
NFXF	National Fragile X Foundation
FRAXA	Fragile X Syndrome Research and Treatment Foundation
FXTAS-RS	Fragile X-associated tremor/ataxia syndrome rating scale
BDI	Beck Depression Inventory
BAI	Beck Anxiety Inventory
CVLT	California Verbal Learning Test
LDFR	Long Delay Free Recall
LDCR	Long Delay Cued Recall
SDFR	Short Delay Free Recall
SDCR	Short Delay Cued Recall
WASI	Wechsler Abbreviated Scale of Intelligence
BDS	Behavioral Dyscontrol Scale
FSIQ	Full Scale IQ
MRI	Magnetic Resonance Imaging
DTI	Diffusion Tensor Imaging
AR	Activation Ratio
Sb	Southern Blot
MVC	Maximum voluntary contraction
CoV	Coefficient of variation
ApEn	approximate entropy
RSPAN	Reading Span
Visual PA	Visual paired associates

## Appendix A

**Table A1:** Correlations between motor and memory measures by group.

Controls	1	2	3	4	5	6	7	8	9
1. Average latency (rise)	-								
2. Average peak velocity	−0.35	-							
3. Accuracy of Initial Force Output (low gain)	−0.08	0.33	-						
4. Sustained accuracy	−0.16	0.25	0.73 **	-					
5. CoV (med gain)	0.50 *	0.04	−0.01	−0.37	-				
6. ApEn	0.23	−0.65 **	−0.24	−0.24	−0.09	-			
7. Visual PA	−0.02	0.13	−0.12	0.01	−0.23	0.22	-		
8. Visual PA RT	0.19	−0.26	−0.31	−0.13	0.09	0.14	−0.09	-	
9. RSPAN	0.06	0.09	0.24	0.28	0.18	−0.25	0.02	−0.03	-
FXTAS−	1	2	3	4	5	6	7	8	9
1. Average latency (rise)	-								
2. Average peak velocity	−0.28	-							
3. Accuracy of Initial Force Output (low gain)	−0.39 *	0.54 **	-						
4. Sustained accuracy	−0.10	0.47 **	0.67 **	-					
5. CoV (med gain)	0.34	−0.39 *	−0.34	−0.43 **	-				
6. ApEn	0.00	−0.22	−0.12	0.02	−0.34	-			
7. Visual PA	−0.16	0.37 *	0.13	0.08	−0.24	0.08	-		
8. Visual PA RT	0.19	0.11	0.21	−0.08	0.03	−0.12	−0.14	-	
9. RSPAN	−0.20	0.19	−0.02	0.11	−0.25	−0.25	0.21	−0.08	-
FXTAS+	1	2	3	4	5	6	7	8	9
1. Average latency (rise)	-								
2. Average peak velocity	−0.39	-							
3. Accuracy of Initial Force Output (low gain)	−0.19	0.34	-						
4. Sustained accuracy	−0.22	0.32	0.91 **	-					
5. CoV (med gain)	0.14	0.02	−0.06	−0.08	-				
6. ApEn	0.08	−0.04	0.16	0.09	−0.69 **	-			
7. Visual PA	0.21	−0.51	−0.48	−0.43	−0.10	0.11	-		
8. Visual PA RT	0.39	−0.10	−0.09	−0.19	−0.04	0.26	−0.41	-	
9. RSPAN	0.30	0.15	−0.23	−0.05	−0.10	−0.20	0.38	−0.22	-

Control participants are not included in comparisons that include CGG repeat or FXTAS-RS. Values are Pearson correlations. Abbreviations: CGG, cytosine-guanine-guanine; FXTAS-RS, FXTAS Rating Scale; Wechsler Abbreviated Scale of Intelligence subtests: FSIQ, full-scale IQ; California Verbal Learning Task subscores: SD FR, short delay free recall; SD CR, short delay cued recall; LD FR, long delay free recall; LD CR, long delay cued recall; BDS-II, Behavioral Dyscontrol Scale; BDI, Beck Depression Inventory; BAI: Beck Anxiety Inventory; Visual PA: visual paired associates accuracy; Visual PA RT: visual paired associates reaction time; RSPAN: reading span accuracy; CoV: Force variability; ApEn: approximate entropy; *p* < 0.05 (*), *p* < 0.01 (**).

**Table A2:** Correlations between motor, genetic, and clinical measures.

Controls	1	2	3	4	5	6	7	8	9	10	11	12	13	14	15	16				
1. Age	-																			
2. Education	−0.09	-																		
3. Average latency (rise)	0.11	−0.17	-																	
4. Average peak velocity	−0.46 *	0.06	−0.35	-																
5. Accuracy of Initial Force Output (low gain)	−0.05	0.17	−0.08	0.33	-															
6. Sustained Accuracy	−0.07	0.30	−0.16	0.25	0.73 **	-														
7. CoV (med gain)	0.13	−0.28	0.50 *	0.04	−0.01	−0.37	-													
8. ApEn	0.10	0.12	0.23	−0.65 **	−0.24	−0.24	−0.09	-												
9. FSIQ	0.47 *	−0.23	0.28	−0.25	−0.19	−0.38	0.35	0.19	-											
10. BDS	−0.16	0.18	−0.09	0.06	−0.01	0.11	−0.16	0.23	−0.32	-										
11. BDI	0.04	0.00	−0.02	0.32	0.13	0.04	0.15	−0.18	0.10	−0.03	-									
12. BAI	0.19	0.01	−0.06	−0.04	−0.05	−0.06	−0.02	−0.12	0.39	0.00	0.55 **	-								
13. LDFR	0.37	0.08	0.04	−0.44 *	−0.48 *	−0.19	−0.24	0.14	−0.01	0.06	−0.45 *	−0.12	-							
14. LDCR	0.18	0.03	0.02	−0.40	−0.48 *	−0.16	−0.44 *	0.25	−0.17	0.27	−0.31	−0.11	0.83 **	-						
15. SDFR	0.30	0.14	−0.04	−0.18	−0.31	0.03	−0.42 *	−0.10	−0.07	0.24	−0.22	−0.04	0.75 **	0.78 **	-					
16. SDCR	0.20	0.02	−0.12	−0.24	−0.41	−0.05	−0.40	−0.13	−0.09	0.17	−0.40	−0.01	0.87 **	0.84 **	0.83 **	-				
FXTAS−	1	2	3	4	5	6	7	8	9	10	11	12	13	14	15	16	17	18	19	20
1. Age	–																			
2. Education	−0.10	–																		
3. Average latency (rise)	0.28	−0.21	–																	
4. Average peak velocity	−0.17	0.01	−0.28	–																
5. Accuracy of Initial Force Output (low gain)	−0.10	0.12	−0.39 *	0.54 **	–															
6. Sustained Accuracy	−0.21	−0.08	−0.10	0.47 *	0.67 **	–														
7. CoV (med gain)	0.39 *	0.14	0.34	−0.39 *	−0.34	−0.43 *	–													
8. ApEn	−0.22	−0.19	0.00	−0.22	−0.12	0.02	−0.34	–												
9. FSIQ	0.09	0.37 *	−0.15	0.30	0.26	0.15	−0.14	−0.12	–											
10. BDS	0.16	0.28	0.05	−0.01	0.07	−0.11	−0.06	−0.01	0.47 **	–										
11. BDI	−0.23	−0.01	0.04	0.03	0.16	0.11	0.00	0.11	0.16	−0.10	–									
12. BAI	−0.22	0.05	−0.23	0.01	−0.04	−0.19	−0.09	0.05	−0.01	−0.14	0.55 **	–								
13. LDFR	0.07	−0.19	−0.12	0.33	0.34 *	0.33	−0.27	−0.09	0.43 *	0.03	0.05	0.15	–							
14. LDCR	0.20	−0.17	−0.07	0.18	0.37 *	0.24	−0.08	−0.13	0.37*	0.08	0.17	0.14	0.86**	–						
15. SDFR	0.15	−0.10	−0.19	0.26	0.32	0.23	−0.22	−0.18	0.40 *	0.06	0.08	0.19	0.92 **	0.83 **	–					
16. SDCR	0.15	−0.05	−0.06	0.23	0.39 *	0.25	−0.11	−0.17	0.38 *	0.06	0.18	0.28	0.88 **	0.89 **	0.87 **	–				
17. RAW CGG	−0.43 **	−0.09	−0.04	0.14	−0.07	0.16	−0.37 *	0.00	0.12	−0.05	−0.06	−0.09	0.16	0.06	0.22	0.12	–			
18. Activation Ratio	−0.29	0.31	−0.15	0.05	0.19	0.30	−0.15	−0.22	−0.13	−0.13	0.12	0.19	0.05	0.12	0.13	0.13	0.16	–		
19. FXTAS-RS	0.40 *	−0.09	0.03	0.04	0.05	0.19	0.29	−0.05	0.06	−0.09	−0.11	−0.16	0.30	0.26	0.28	0.32	−0.13	−0.08	–	
20. CGGobs	−0.05	−0.34	0.08	−0.02	−0.22	−0.19	−0.07	0.22	0.15	0.02	−0.12	−0.23	0.14	0.03	0.09	0.04	0.48 *	−0.73 **	0.02	–
FXTAS+	1	2	3	4	5	6	7	8	9	10	11	12	13	14	15	16	17	18	19	20
1. Age	–																			
2. Education	−0.08	–																		
3. Average latency (rise)	−0.10	0.60 *	–																	
4. Average peak velocity	−0.23	−0.26	−0.39	–																
5. Accuracy of Initial Force Output (low)	−0.06	−0.28	−0.19	0.34	–															
6. Sustained Accuracy	−0.03	−0.12	−0.22	0.32	0.91 **	–														
7. CoV (med)	0.12	0.02	0.14	0.02	−0.06	−0.08	–													
8. ApEn	−0.21	0.12	0.08	−0.04	0.16	0.09	−0.69 **	–												
9. FSIQ	0.32	0.31	−0.09	−0.36	−0.24	−0.01	0.26	−0.19	–											
10. BDS	−0.28	0.39	0.61 *	−0.41	−0.36	−0.30	0.04	0.32	0.15	–										
11. BDI	−0.37	−0.25	−0.13	0.02	0.09	0.05	−0.43	0.20	−0.43	−0.06	–									
12. BAI	−0.16	−0.34	−0.29	0.23	0.02	−0.03	−0.05	−0.27	−0.39	−0.32	0.80 **	–								
13. LDFR	−0.01	0.10	0.09	−0.02	−0.34	−0.29	−0.40	0.19	0.08	0.25	0.31	0.15	–							
14. LDCR	0.11	0.23	0.06	−0.05	−0.16	−0.02	−0.45	0.21	0.30	0.22	0.16	−0.04	0.89 **	–						
15. SDFR	−0.14	0.24	0.25	0.11	−0.41	−0.44	−0.10	−0.02	0.07	0.13	−0.06	−0.08	0.76 **	0.66 **	–					
16. SDCR	−0.11	0.39	0.32	−0.20	−0.33	−0.35	−0.32	0.29	0.05	0.42	0.15	0.06	0.74 **	0.71 **	0.70 **	–				
17. RAW CGG	−0.18	−0.05	−0.32	0.39	0.18	0.33	−0.08	0.01	−0.12	−0.06	0.03	−0.01	0.14	0.16	0.00	−0.07	–			
18. Activation Ratio	0.11	−0.10	0.31	−0.26	−0.09	−0.10	0.13	−0.34	−0.19	0.15	0.21	0.40	0.11	0.06	0.07	0.38	−0.11	–		
19. FXTAS-RS	0.19	−0.37	−0.10	0.46	0.49	0.31	0.33	−0.04	−0.45	−0.17	0.11	0.23	−0.15	−0.22	−0.22	−0.26	0.20	−0.05	–	
20. CGGobs	−0.19	−0.01	−0.38	0.45	0.32	0.41	−0.20	0.34	0.04	−0.15	−0.12	−0.34	−0.09	0.00	−0.17	−0.43	0.66 **	−0.78 **	0.18	–

Control participants are not included in comparisons that include CGG repeat or FXTAS-RS. Values are Pearson correlations. Abbreviations: CGG, cytosine-guanine-guanine; FXTAS-RS, FXTAS Rating Scale; Wechsler Abbreviated Scale of Intelligence subtests: FSIQ, full-scale IQ; California Verbal Learning Task subscores: SD FR, short delay free recall; SD CR, short delay cued recall; LD FR, long delay free recall; LD CR, long delay cued recall; BDS-II, Behavioral Dyscontrol Scale; BDI, Beck Depression Inventory; BAI: Beck Anxiety Inventory; CoV: Force variability; ApEn: approximate entropy; *p* < 0.05 (*), *p* < 0.01 (**).

**Table A3:** Correlations between memory, genetic, and clinical measures.

Controls	1	2	3	4	5	6	7	8	9	10	11	12	13				
1. Age	-																
2. Education	−0.09	-															
3. Visual PA	−0.36	0.00	-														
4. Visual PA RT	0.43 *	0.07	−0.09	-													
5. RSPAN	−0.12	−0.22	0.02	−0.03	-												
6. FSIQ	0.47 *	−0.23	−0.13	0.11	0.27	-											
7. BDS	−0.16	0.18	0.34	−0.14	−0.02	−0.32	-										
8. BDI	0.04	0.00	−0.52 *	0.02	−0.11	0.10	−0.03	-									
9. BAI	0.19	0.01	−0.61 **	0.01	0.05	0.39	0.00	0.55 **	-								
10. LDFR	0.37	0.08	0.07	0.42 *	0.01	−0.01	0.06	−0.45 *	−0.12	-							
11. LDCR	0.18	0.03	0.18	0.33	−0.14	−0.17	0.27	−0.31	−0.11	0.83 **	-						
12. SDFR	0.30	0.14	0.08	0.22	0.03	−0.07	0.24	−0.22	−0.04	0.75 **	0.78 **	-					
13. SDCR	0.20	0.02	0.14	0.18	0.11	−0.09	0.17	−0.40	−0.01	0.87 **	0.84 **	0.83 **	-				
FXTAS−	1	2	3	4	5	6	7	8	9	10	11	12	13	14	15	16	17
1. Age	-																
2. Education	−0.10	-															
3. Visual PA	−0.19	0.04	-														
4. Visual PA RT	0.26	0.03	−0.14	-													
5. RSPAN	0.01	0.12	0.21	−0.08	-												
6. FSIQ	0.09	0.37 *	0.42 *	0.09	0.20	-											
7. BDS	0.16	0.28	0.34	0.00	0.18	0.47 **	-										
8. BDI	−0.23	−0.01	0.26	−0.06	−0.07	0.16	−0.10	-									
9. BAI	−0.22	0.05	0.05	0.19	−0.06	−0.01	−0.14	0.55 **	-								
10. LDFR	0.07	−0.19	0.20	0.22	0.20	0.43 *	0.03	0.05	0.15	-							
11. LDCR	0.20	−0.17	0.15	0.25	0.24	0.37 *	0.08	0.17	0.14	0.86 **	-						
12. SDFR	0.15	−0.10	0.17	0.20	0.19	0.40 *	0.06	0.08	0.19	0.92 **	0.83 **	-					
13. SDCR	0.15	−0.05	0.17	0.30	0.20	0.38 *	0.06	0.18	0.28	0.88 **	0.89 **	0.87 **	-				
14. RAW CGG	−0.43 **	−0.09	0.09	−0.15	0.11	0.12	−0.05	−0.06	−0.09	0.16	0.06	0.22	0.12	-			
15. Activation Ratio	−0.29	0.31	−0.20	0.04	0.25	−0.13	−0.13	0.12	0.19	0.05	0.12	0.13	0.13	0.16	-		
16. FXTAS-RS	0.40 *	−0.09	−0.05	−0.01	−0.05	0.06	−0.09	−0.11	−0.16	0.30	0.26	0.28	0.32	−0.13	−0.08	-	
17. CGGobs	−0.05	−0.34	0.22	−0.19	−0.16	0.15	0.02	−0.12	−0.23	0.14	0.03	0.09	0.04	0.48 **	−0.73 **	0.02	-
FXTAS+	1	2	3	4	5	6	7	8	9	10	11	12	13	14	15	16	17
1. Age	-																
2. Education	−0.08	-															
3. visual PA	−0.25	0.45	-														
4. visual PA RT	0.29	−0.14	−0.41	-													
5. RSPAN	−0.30	0.36	0.38	−0.22	-												
6. FSIQ	0.32	0.31	0.21	0.00	−0.16	-											
7. BDS	−0.28	0.39	0.52 *	0.35	0.15	0.15	-										
8. BDI	−0.37	−0.25	0.05	−0.30	−0.17	−0.43	−0.06	-									
9. BAI	−0.16	−0.34	−0.17	−0.34	−0.14	−0.39	−0.32	0.80 **	-								
10. LDFR	−0.01	0.10	0.35	−0.06	0.21	0.08	0.25	0.31	0.15	-							
11. LDCR	0.11	0.23	0.40	−0.06	0.20	0.30	0.22	0.16	−0.04	0.89 **	-						
12. SDFR	−0.14	0.24	0.29	−0.03	0.31	0.07	0.13	−0.06	−0.08	0.76 **	0.66 *	-					
13. SDCR	−0.11	0.39	0.54 *	0.02	0.20	0.05	0.42	0.15	0.06	0.74 **	0.71 **	0.70 **	-				
14. RAW CGG	−0.18	−0.05	−0.05	−0.48	0.23	−0.12	−0.06	0.03	−0.01	0.14	0.16	0.00	−0.07	-			
15. Activation Ratio	0.11	−0.10	0.06	0.22	0.21	−0.19	0.15	0.21	0.40	0.11	0.06	0.07	0.38	−0.11	-		
16. FXTAS-RS	0.19	−0.37	−0.40	0.02	−0.36	−0.45	−0.17	0.11	0.23	−0.15	−0.22	−0.22	−0.26	0.20	−0.05	-	
17. CGGobs	−0.19	−0.01	−0.25	−0.25	−0.13	0.04	−0.15	−0.12	−0.34	−0.09	0.00	−0.17	−0.43	0.66 *	−0.78 *	0.18	-

Control participants are not included in comparisons that include CGG repeat or FXTAS-RS. Values are Pearson correlations. Abbreviations: CGG, cytosine-guanine-guanine; FXTAS-RS, FXTAS Rating Scale; Wechsler Abbreviated Scale of Intelligence subtests: FSIQ, full-scale IQ; California Verbal Learning Task subscores: SD FR, short delay free recall; SD CR, short delay cued recall; LD FR, long delay free recall; LD CR, long delay cued recall; BDS-II, Behavioral Dyscontrol Scale; BDI, Beck Depression Inventory; BAI: Beck Anxiety Inventory; Visual PA: visual paired associates accuracy; Visual PA RT: visual paired associates reaction time; RSPAN: reading span accuracy; *p* < 0.05 (*), *p* < 0.01 (**).
